# Medical Men as Coroners

**Published:** 1864-11

**Authors:** 


### EDITORIAL DEPARTMENT.

### MEDICAL MEN AS CORONERS.

It would seem that the principle would long since have been established that this office belongs by right exclusively to the medical profession; indeed it is strange that it should ever have been regarded otherwise, for no one without a medical education is competent to perform its duties, let him be ever so intelligent in other respects. It is not however as yet a recognized necessity, and it remains for the profession to exercise their influence in directing the public mind and endeavoring to awaken it to a just appreciation of the subject. Were the public to fully understand the functions of the coroner, and to reflect how greatly the due administration of justice in criminal cases depends upon the examinations and investigations of this officer, none other than an intelligent physician would ever be proposed for this duty. Attention has been sufficiently called to this matter it would seem, but the ignorant and unqualified are more clamorous for place than any other, and the examination of the most intricate questions, involving character and life, is still committed to the jurisdiction of men profoundly ignorant of everything relating to these questions. The public have not yet come to see the importance and necessity of placing medical men in office as coroner, partly because they do not understand that the duties of this office cannot be discharged with ability by any except those possessed of thorough medical education. We desire to make the point plain, first to the profession, and through it to the public, that a coroner should be an intelligent physician, and that no others are, or can be suitable for such office.

We have prepared quite a *resumé* of the duties which legitimately belong to this office, and have pointed out some of the abuses and misapprehensions which we believe are common in connection with it. But upon reflection we have set it aside for the present, since it is a thankless and unpleasant task to expose or correct abuses unless it be made in some way our especial duty, and we do not as yet see that the obligation rests with us.

There is one point upon which we cannot withhold a remark, though perhaps it can be in no way productive of reform. When it is necessary to make inquest in cases of suspected crime, it should be done thoroughly, impartially, and scientifically, and when physicians are called to perform *post mortem* examinations with view of obtaining positive testimony, or when microscopic and chemical examinations are made with similar object, the compensation should be adequate, sufficient to secure the highest talent, and command the most thorough investigation. The Boston fee bill for physicians, places the price of *post mortem* examinations and testimony before the coroner at $50, while Erie County Supervisors place it at $10, and the Buffalo Medical Association in its old fee bill, placed it at that price; it has never been changed.

The election returns which we stop writing to examine, show the probable election of another medical man to the office of coroner in this city, so that now we may be said to have gained our point. We have gained it in fact, but not in principle. The party which has placed two regular physicians in office as Coroner, is more likely at the next election to place some ex-Justice of the Peace or ex-constable in their places, upon the principle of "rotation in office" than to re-elect them or others qualified for its duties. The ground of claim to this position is wholly misapprehended by the political masses who apportion this place with the other places in their gift, to those who seek them the most constantly, or call for them the most loudly. Whatever be the circumstances and influences of election the profession look for correction of abuses to the physicians who are now holding, or are soon to hold these offices. And though their places will be bought and sold by political huxters, they may yet leave an example which others will follow "which it will be dangerous for their successors to neglect." It is the duty of the profession to use its influence in opposition to the nomination, and if unsuccessful in this, to the election of any other than intelligent physicians, to this office. We believe that united effort in this matter, is controlling—that the "balance of power" is with us.

